# Embodying the Aging Experience: How Virtual Reality Is Transforming Medical and Nursing Education

**DOI:** 10.1093/geroni/igab046.232

**Published:** 2021-12-17

**Authors:** Marilyn Gugliucci, Pamela Saunders, Erin Washington

## Abstract

Virtual reality (VR) has long been standard in healthcare education. Recent advances in VR hardware and software applications have coalesced to allow for higher fidelity, more highly realistic simulations that are also deployable at scale -- not just in highly specialized, single location simulation labs. In tandem, there has been an examination in both the corporate and academic sectors around the efficacy of VR training and learning. While VR has been long proven to be effective in training students and workers in hard skills, its lack of realism has been a barrier to explore efficacy in simulations related to soft skills and emotional intelligence. This symposium will discuss the implementation of virtual reality “labs”, where learners embody in a live 360 film environment the first-person point of view of an older adult -- interacting with gaze, voice, and natural hand motions – into four university’s medical and nursing curriculum. Lab outcomes include decreased ageism and stereotyping, and increased empathy, sensitivity, cultural competency, and disease knowledge. The first paper reports outcomes of increased understanding, comfort, compassion and empathy of students and informal caregivers after experiencing various labs. The second discusses comparative data on knowledge and attitudes of medical students experiencing the virtual labs individually vs. the group distance mode. The third reports the results of an initial study on how embodying an older adult with sensory impairment affects participant empathy using a standardized scale. The fourth discusses how one university transitioned to delivering immersive labs to nursing students remotely during COVID19.

